# Two-Dimensional Materials: From Synthesis to Applications, 2nd Edition

**DOI:** 10.3390/molecules31020215

**Published:** 2026-01-08

**Authors:** Sake Wang, Nguyen Tuan Hung, Minglei Sun

**Affiliations:** 1 College of Science, Jinling Institute of Technology, 99 Hongjing Avenue, Nanjing 211169, China; 2 Frontier Research Institute for Interdisciplinary Sciences, Tohoku University, Sendai 980-8578, Japan; nguyen.tuan.hung.e4@tohoku.ac.jp; 3 NANOlab Center of Excellence and Department of Physics, University of Antwerp, Groenenborgerlaan 171, 2020 Antwerp, Belgium; minglei.sun@uantwerpen.be

## 1. Introduction

Two-dimensional materials continue to redefine modern materials science by offering a unique combination of atomic-scale thickness, tunable electronic structures, and highly accessible surfaces [1]. Since the emergence of graphene, the field has expanded dramatically to include elemental 2D materials, transition-metal dichalcogenides, nitrides, oxides, and a wide variety of hybrid and composite systems [2]. These materials have enabled conceptual and technological breakthroughs across nanoelectronics, optoelectronics [3], energy storage, catalysis, sensing, and environmental remediation [4].

Building upon the first edition [5], the second edition of the Special Issue “Two-Dimensional Materials: From Synthesis to Applications” in *Molecules* captures this breadth and maturity of the field. The contributions span theoretical modeling, controlled synthesis, heterostructure engineering, device design, catalytic mechanisms, and advanced characterization methodologies [6]. Collectively, the papers illustrate how fundamental understanding at the atomic and electronic levels translates into real-world applications.

Notably, the contributors to this Special Issue come from Bulgaria, China, Poland, Russia, and the USA, highlighting the global and collaborative nature of contemporary research in low-dimensional materials.

## 2. Scope and Thematic Organization of the Special Issue

To provide a coherent overview, the eight contributions can be broadly classified into four interrelated thematic categories:

1. Fundamental Physics and Electronic Structure of 2D Materials (Paper [7]).
2. Two-Dimensional Materials and Heterostructures for Optoelectronic and Electromagnetic Devices (Papers [8,9]).
3. Energy Storage and Conversion Enabled by 2D and 2D-Derived Materials (Papers [10,11]).
4. Catalysis, Environmental Remediation, and Advanced Characterization (Papers [12,13,14]).

Table 1 summarizes the thematic classification, material systems, and targeted applications of each contribution.

## 3. Fundamental Physics and Electronic Properties of Low-Dimensional Systems

A defining strength of 2D materials lies in their sensitivity to reduced dimensionality, edges, defects, and external perturbations. Rakshit et al. [7] present a comprehensive first-principles investigation of α-borophene nanoribbons, a 1D derivative of a metallic 2D boron sheet. Their work demonstrates how edge geometry (armchair vs. zigzag), ribbon width, and edge termination collectively determine electronic conduction and magnetism.

Of particular significance is the emergence of edge magnetism and spin-polarized transport in zigzag nanoribbons, with spin filtering efficiencies exceeding 40%. Such findings place borophene-based nanostructures at the forefront of spintronic interconnects, where metallic conductivity and tunable magnetism must coexist. This study exemplifies how theoretical modeling can guide the rational design of low-dimensional components for future nanoelectronic circuits.

## 4. Two-Dimensional Materials for Optoelectronic and Electromagnetic Devices

The integration of 2D materials into functional devices is strongly represented in this Special Issue. Zhou et al. [8] report a ZnO–MoS_2_ type-II heterostructure [15,16] photodetector with an exceptionally broad operational range spanning from ultraviolet to MIR wavelengths (365 nm–10 µm). The combination of experimental device fabrication and DFT calculations reveals how defect engineering—specifically Mo vacancies—reduces the effective bandgap to ~0.20 eV, enabling MIR detection.

Equally compelling is the demonstration of self-powered operation, fast response times, and light-controlled hysteresis, which opens avenues for optical memory and neuromorphic computing. This work highlights the versatility of 2D heterostructures in multifunctional optoelectronic platforms [3].

Complementing photodetection, Zhou et al. [9] explore THz absorption using hybrid systems composed of graphene and bulk Dirac semimetals. Through electromagnetic simulations and impedance-matching analysis, they demonstrate broadband absorption exceeding 80% in the 7.7–9.2 THz range. The dual tunability arising from graphene and Dirac semimetals underscores how 2D materials can be engineered for adaptive electromagnetic environments, relevant to sensing, communication, and stealth technologies.

## 5. Energy Storage: 2D Materials in Lithium-Ion Batteries

Energy storage remains a central challenge for sustainable technologies, and several contributions address this domain. Wei et al. [10] introduce a general synthesis route to ZnS nanoparticles embedded in 2D carbon films, derived from the thermal decomposition of organic zinc salts. The resulting ZnS/C composites show excellent lithium storage performance, benefiting from enhanced electrical conductivity and effective buffering of volume changes during lithiation.

Wu et al. [11] further advance this theme by designing Co-doped GaN nanowires grown directly on carbon paper. By combining experimental electrochemical measurements with DFT calculations, the authors reveal how Co^2+^ substitution modifies the electronic structure of GaN, lowers ion diffusion barriers, and significantly improves cycling stability. The work exemplifies how doping strategies and hierarchical architectures can transform traditionally inert semiconductors into high-performance battery electrodes.

Together, these studies demonstrate that 2D and quasi-2D materials are not merely passive hosts but active participants in charge storage and transport processes.

## 6. Catalysis, Environmental Remediation, and Surface Phenomena

Catalytic and environmental applications are another strong pillar of this Special Issue. Ivanova et al. [12] introduce an innovative tribocatalytic approach using ZnO and ZnO/Eu_2_O_3_ sol–gel catalysts, where mechanical friction replaces light as the driving energy source. The ability to degrade paracetamol under dark conditions highlights a promising pathway for green chemistry and sustainable pollutant removal, leveraging ubiquitous mechanical energy in natural environments.

In a complementary photocatalytic context, Feng et al. [13] investigate Pt-decorated SnS_2_ nanosheets for tetracycline degradation. The synergy between sheet-like SnS_2_ morphology and the LSPR of Pt nanoparticles leads to enhanced light absorption, efficient charge separation, and high degradation efficiency. This work reinforces the importance of metal–2D semiconductor interfaces in solar-driven environmental remediation.

## 7. Advanced Characterization and Conceptual Expansion Beyond Strictly 2D Systems

While this Special Issue focuses on 2D materials, Shelyapina [14] provides a timely and insightful review on NMR relaxation techniques for probing zeolites. Although zeolites are not strictly 2D materials, their hierarchical porosity, surface chemistry, and confined diffusion phenomena resonate strongly with concepts central to 2D materials science.

The review highlights how 2D NMR relaxation methods ($T_{1}$–$T_{2}$ and $T_{2}$–$T_{2}$ correlations) enable unprecedented insight into molecular mobility, surface acidity, pore size distribution, and connectivity. These techniques are increasingly relevant for characterizing 2D-based catalysts, membranes, and hybrid porous systems, thereby broadening the methodological toolkit available to the community.

## 8. Outlook and Perspectives

The contributions collected in this second edition collectively demonstrate that the field of 2D materials has progressed from isolated material discovery toward system-level integration, multifunctionality, and real-world relevance [17]. From edge-engineered borophene nanoribbons and defect-tuned heterostructures to mechanically driven catalysis and advanced spectroscopic characterization, the Special Issue reflects both depth and diversity.

This edition will stimulate continued interdisciplinary collaboration, inspiring both curiosity and application-driven exploration in materials science, which we sincerely hope will contribute to the next edition of this Special Issue, “Two-Dimensional Materials: From Synthesis to Applications, 3rd Edition”.

## Figures and Tables

**Table 1:** Overview and categorization of contributions in this Special Issue.

Ref.	Material System	Dimensionality/Structure	Main Focus	Key Application
[7]	α-Borophene nanoribbons	1D ribbons from 2D sheet	Edge-dependent electronic and magnetic properties	Nanoelectronics, spintronics
[8]	ZnO–MoS_2_ heterostructure	2D/2D p–n junction	Ultra-broadband photodetection	Optoelectronics, internet of things sensing
[9]	Graphene + Dirac semimetals	Hybrid layered structures	Tunable THz absorption	Electromagnetic devices
[10]	ZnS/2D carbon films	2D carbon-supported nanocomposites	Lithium storage mechanisms	Li-ion batteries
[11]	Co-doped GaN nanowires	1D/2D hybrid architecture	Doping-enhanced electrochemistry	Li-ion batteries
[12]	ZnO, ZnO/Eu_2_O_3_	Nanostructured oxide catalysts	Tribocatalysis driven by mechanical energy	Environmental remediation
[13]	Pt–SnS_2_ nanosheets	2D semiconductor/metal hybrids	LSPR-assisted photocatalysis	Water purification
[14] (review)	Zeolites	Micro-/mesoporous frameworks	NMR relaxation characterization	Catalysis, porous materials

## Abbreviations

The following abbreviations are used in this manuscript:

1D	one-dimensional
2D	two-dimensional
DFT	density functional theory
LSPR	local surface plasmon resonance
MIR	mid-infrared
NMR	nuclear magnetic resonance
THz	terahertz
